# Prognostic Impact of Tumor Solid Components in Stereotactic Body Radiotherapy for Clinical Stage Tis–1N0M0 Lung Cancer

**DOI:** 10.1111/1759-7714.70110

**Published:** 2025-06-16

**Authors:** Junki Fukuda, Hiroshi Doi, Atsushi Kono, Takaya Inagaki, Naoko Ishida Hamazawa, Saori Tatsuno Imamura, Takuya Uehara, Masahiro Inada, Kiyoshi Nakamatsu, Makoto Hosono, Kazunari Ishii, Yukinori Matsuo

**Affiliations:** ^1^ Department of Radiation Oncology Kindai University Faculty of Medicine Osaka‐Sayama Japan; ^2^ Department of Radiology Kindai University Faculty of Medicine Osaka‐Sayama Japan; ^3^ Department of Radiology Wakayama Medical University Wakayama Japan

**Keywords:** consolidation tumor ratio, ground‐glass opacity, lung cancer, stereotactic ablative radiotherapy, stereotactic body radiation therapy

## Abstract

**Purpose:**

This study aimed to assess the potential of prognostic factors including consolidation tumor ratio (CTR) on treatment outcomes in patients with clinical stage 0–IA non‐small cell lung cancer (NSCLC) undergoing stereotactic body radiotherapy (SBRT).

**Methods:**

The analysis included data of 63 patients with 67 lesions of clinical stage 0–IA NSCLC treated with SBRT. According to the Union for International Cancer Control 8th edition, the following tumor stages were observed: Tis, 3; T1mi, 2; T1a, 11; T1b, 29; and T1c, 22. The prescribed dose was 48 (range, 42–52) Gy in four fractions.

**Results:**

The median follow‐up was 29.3 (range: 2.4–120.5) months. The five‐year local control (LC), overall survival, and progression‐free survival (PFS) rates were 89.4%, 60.3%, and 40.5%, respectively. Squamous cell carcinoma (Sq) and D_max_ < 125 Gy_BED10_ for planning target volume (PTV) were associated with a worse LC (*p* = 0.001 and 0.017, respectively). Patients with Sq, T1b–c, CTR > 0.25, PTV ≥ 30 cm^3^ tumors were associated with worse PFS than those with non‐Sq, ≤ cT1a, CTR ≤ 0.25, PTV < 30 cm^3^ tumors (*p* = 0.049, 0.004, 0.038, and 0.004, respectively). No recurrences, metastases, or deaths were found in patients with CTR ≤ 0.25 (*n* = 5).

**Conclusion:**

In patients with stage 0–IA lung cancer treated with SBRT, tumors classified as ≤ T1a showed a better PFS than T1b–c. NSCLC with a low CTR of ≤ 0.25 seemed to have a low risk of recurrence after SBRT.

## Graphical abstract

Progression‐free survival (PFS) per T stage and consolidation tumor ratio (CTR) in all eligible patients.

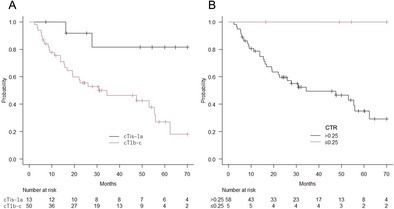

- PFS for patients per T stage. Patients with tumors of cT1a or less showed significantly better PFS than those with cT1b or c (*n* = 13 and 50 in cT1a or less and cT1b and c groups, respectively; *p* = 0.004).
- PFS for patients per CTR. Patients with tumors of CTR ≤ 0.25 showed significantly better PFS than those with > 0.25 (*n* = 5 and 58 in CTR ≤ 0.25 and > 0.25 groups; *p* = 0.038).

## 1 Introduction

The management of early‐stage non‐small cell lung cancer (NSCLC) has developed significantly with advancements in diagnostic imaging, radiotherapy, and surgical techniques. The inclusion of ground‐glass opacity (GGO) components in staging, first introduced in the 8th edition of the Union for International Cancer Control (UICC) TNM classification, marked a turning point in lung cancer stratification over UICC 7th edition in which the tumor size included GGO [1, 2]. This staging revision acknowledged the prognostic value of GGO and consolidation tumor ratio (CTR), a metric reflecting the proportion of solid tumor components. Tumors with low CTR (e.g., CTR < 0.25) are typically associated with less invasive adenocarcinomas, such as adenocarcinoma in situ or minimally invasive adenocarcinoma, and have more favorable prognosis [3, 4]. Building upon these insights, the 8th edition of the UICC TNM classification further refined staging criteria, emphasizing the role of CTR in distinguishing tumor biology and guiding treatment strategies.

In surgical practice, CTR has been widely studied as a prognostic indicator, with established evidence supporting its role in determining the extent of resection. Tumors with CTR < 0.25 are often treated with wedge resection, whereas those with CTR of 0.26–0.5 are candidates for segmentectomy, with both approaches yielding excellent outcomes in terms of local recurrence and survival [5, 6]. Tumors, characterized by low CTR, are associated with indolent behavior and favorable prognosis, raising questions about the optimal treatment approach [7, 8].

Stereotactic body radiotherapy (SBRT) has emerged as a standard treatment in patients with medically inoperable early‐stage NSCLC, offering excellent local control (LC) and potential survival outcomes with minimal toxicity [9, 10, 11]. Several prognostic factors have been reported to influence prognosis in patients with lung cancer who underwent SBRT [12, 13, 14, 15, 16, 17, 18, 19, 20, 21]. In addition, we have previously reported that pathologically confirmed squamous cell carcinoma (Sq), maximum dose (D_max_) for PTV < 125 Gy (BED_10_), and cT2 stage were associated with poorer LC [20]. However, cTis or cT1 (cTis/1) N0M0 tumors, meaning clinical stage 0–IA according to the UICC 8th edition, comprise heterogeneous tumor types, such as pathological subtypes, gene expression, and CTR rate. In addition, the application of SBRT to biologically favorable tumors in patients with cTis or T1 tumors is not fully understood [22, 23, 24, 25, 26]. The aim of the present study was to assess the potential prognostic factors including CTR in patients with clinical T1 (cT1) or less (Stage 0–IA) NSCLC.

## 2 Methods

This retrospective study was approved by the Ethics Committee of Kindai University, Faculty of Medicine (approval no.: R06‐173). Written informed consent to SBRT was obtained from all individual patients prior to radiotherapy. Informed consent for participating in this study was obtained in the form of opt‐out. This study was conducted in accordance with the principles of the Declaration of Helsinki.

### 2.1 Patient Characteristics

Clinical diagnosis and indications for SBRT were decided based on clinical information, images, and pathological diagnosis by the Thoracic Tumor Board Conference of the Kindai University Hospital (Osaka, Japan). The panel consisted of pulmonologists, medical oncologists, thoracic surgeons, radiation oncologists, and radiologists. Generally, a tumor that showed a continuous increase in size or CTR and positive accumulation on fluorodeoxyglucose‐positron emission tomography (FDG‐PET) was considered a clinically diagnosed primary lung cancer, according to the inclusion criteria of the Japan Clinical Oncology Group Study (JCOG) 1408 study [27].

A total of 172 consecutive patients with 190 lesions who received SBRT for early‐stage lung cancer at Kindai University Hospital between January 2008 and December 2023 were included in the present study. We revised the clinical stage in all patients based on the 8th edition of the UICC [1]. Clinical T3 was diagnosed by evaluating chest wall invasion using four‐dimensional computed tomography (4D‐CT) [21]. Clinical staging and CTR were defined by a radiation oncologist with five years of experience (J.F.) and a radiologist with 22 years of experience (A.K.), who were blinded to the clinical outcomes, according to the UICC 8th edition using high‐resolution computed tomography (HRCT) [1]. The window settings for evaluation of the lung tumors were window‐width 1500 HU and window‐level − 600 HU. Multifocal tumors in a single patient were classified according to the T category of the lesion with the highest T. Patients with pathologically confirmed small cell lung cancer, with cT2 or cT3 tumors according to UICC 8th edition, without available data of HRCT, and who had a follow‐up duration of less than 6 months without any specific events were excluded from this study.

In total, 63 patients with 67 lesions of clinical stage 0–IA lung cancer were evaluated in this retrospective study. Patient characteristics and the flowchart of patient selection are summarized in Table 1 and Supporting Information 1.

**TABLE 1:** Patient characteristics.

Patients	*n* = 63	(%)
Age (y), median (range)	77 (60–88)	
Gender		
Male	38	60.3%
Female	25	39.7%
ECOG‐PS		
0	29	46.0%
1	20	31.7%
2	10	15.9%
3	3	4.8%
4	1	1.6%
Tobacco‐smoking history		
Yes	51	81.0%
Brinkman Index (*n* = 51)	900 (0–3000)	
No	12	19.0%

Tumors	*n* = 67	(%)
Resectability		
Yes	18	26.9%
No (medically inoperable)	49	73.1%
Treatment site		
Upper left	20	29.9%
Lower left	3	4.5%
Upper right	15	22.4%
Middle right	7	10.4%
Lower right	22	32.8%
Pathological diagnosis		
Adenocarcinoma	18	26.9%
Squamous cell carcinoma	12	17.9%
Other	2	3.0%
Unknown	35	52.2%
Clinical T stage		
Tis	3	4.5%
T1mi	2	3.0%
T1a	11	16.4%
T1b	29	43.3%
T1c	22	32.8%
CTR		
≤ 0.25	5	7.5%
> 0.25, ≤ 0.50	4	6.0%
> 0.50	58	86.6%

Radiotherapy	*n* = 67	
CTV margin		
Yes	32	47.8%
No	35	52.2%
Technique		
3D‐CRT	26	38.8%
VMAT	41	61.2%
Prescription doses and fractionations of SBRT		
48 Gy in four fractions	44	65.7%
52 Gy in four fractions	21	31.3%
55 Gy in four fractions	2	3.0%
Dosimetric parameters for PTV		
Volume (cm^3^)	29.6 (4.3–91.3)	
D_max_ (BED_10_, Gy)	152.9 (106.4–197.5)	
D_50%_ (BED_10_, Gy)	119.3 (96.5–160.6)	
D_95%_ (BED_10_, Gy)	105.6 (88.9–130.7)	
D_98%_ (BED_10_, Gy)	101.1 (87.7–126.9)	
D_min_ (BED_10_, Gy)	91.0 (67.3–111.5)	

Abbreviations: 3D‐CRT, three‐dimensional conformal radiotherapy; BED, biologically effective dose; CTR, consolidation tumor ratio; CTV, clinical target volume; D_max_, the maximum dose; D_min_, the minimum dose; D_xx%_, the minimum dose received by XX% of the total volume of PTV; ECOG‐PS, Eastern Cooperative Oncology Group performance status; SBRT, stereotactic body radiation therapy; VMAT, volumetric modulated arc therapy.

### 2.2 Radiotherapy

Radiotherapy for tumors was performed as previously described [20, 21]. Respiratory motion was generally evaluated using 4D‐CT. If respiratory motion of less than 10 mm was expected, the internal target volume (ITV)‐based approach was applied under free‐breathing conditions. If respiratory motion of ≥ 10 mm was expected, the exhalation breath‐hold technique was applied. Briefly, the clinical target volume (CTV) was created from the gross tumor volume (GTV) by adding 5–8 mm margins between January 2008 and March 2018; the CTV was equal to the GTV from March 2018 onwards. As necessary, the ITV was created from the CTV by adding a sufficient margin on 4D‐CT. The PTV was calculated by adding a margin of 5 mm to each CTV or ITV. SBRT was performed using three‐dimensional conformal radiation therapy (3D‐CRT) or volumetric modulated arc therapy (VMAT), which typically used 6 MV X‐rays. SBRT was performed using a VMAT technique from October 2015 onwards due to the replacement of the linear accelerator. The prescribed dose was calculated for delivery to a reference point in 3D‐CRT and was normalized to 95% of the PTV using VMAT. The dose distributions were calculated using the analytical anisotropic (AAA, Eclipse) or Acuros XB (Eclipse) algorithms. The techniques of delivered radiotherapy, prescription dose, and dosimetric parameters in the dose‐volume histogram (DVH) parameters are presented in Table 1. The median prescribed dose was 48 (range, 42–52) Gy in four fractions. Considering the different number of fractions, we thought it would be reasonable to calculate and compare each DVH parameter to the biologically effective dose (BED) using the linear‐quadratic model. The calculation formula for BED was as follows:

$$\mathrm{BED}=n\times d\times \left[1+d/\left(\alpha /\beta \right)\right]$$

where *n* is the number of fractions, *d* is the dose of one fraction, and the value of α/β is 10 Gy for the tumors [28].

### 2.3 Statistical Analysis

The data are expressed as medians with the range in parentheses, unless otherwise indicated. Follow‐up visits, including chest CT, were performed at least once every 3 months up to 2 years after SBRT and at least once every 6 months thereafter. The time to a specific event was defined as the interval from the start of radiotherapy to the date of the event. LC for each tumor was measured from the start of SBRT until recurrence within the PTV. Local recurrence was defined as continuous enlargement in tumor size or abnormal fluorodeoxyglucose uptake that was judged to be viable tumor and not inflammatory change by the diagnostic radiologist. Pathologic confirmation was not mandatory. Overall survival (OS) was measured from the start of SBRT until death from any cause (censored at the date of last confirmed survival for surviving patients). Progression‐free survival (PFS) was measured from the start of SBRT until the first event of disease progression or death, whichever occurred first (censored at the date of last confirmed survival for patients with no events). Disease‐specific survival (DSS) was measured from the start of SBRT until death from disease progression. OS, PFS, and DSS were measured for each patient. The earliest day of the start of SBRT was defined as the starting point of OS, PFS, and DSS in patients who received SBRT twice. The start of SBRT for tumors with more advanced T stage was defined as the starting point of OS, PFS, and DSS in patients diagnosed with two primary tumors of cT1a and cT1b at the same time. Cumulative time was calculated using the Kaplan–Meier method, and the differences in probability curves were assessed using the log‐rank test. The cut‐off values of D_max_ value of 125 Gy (BED_10_) for PTV was decided as previously reported [20]. The cut‐off value of a CTR of 0.25 was decided according to previous reports [5, 29]. The other cut‐off values of possible potential predictive factors were decided based on the median values. The results were reported as hazard ratios with corresponding 95% confidence intervals (CI). The threshold for statistical significance was *p* < 0.05. Variables with *p* < 0.05 from univariate analysis were fitted into a multivariate model using Cox regression analysis. The relationship between the groups was assessed using a two‐tailed Fisher's exact test. Toxicity was graded according to the Common Terminology Criteria for Adverse Events (CTCAE), version 5.0 [30]. All statistical analyses were performed using EZR software (version 4.3.1).

## 3 Results

**FIGURE 1:**
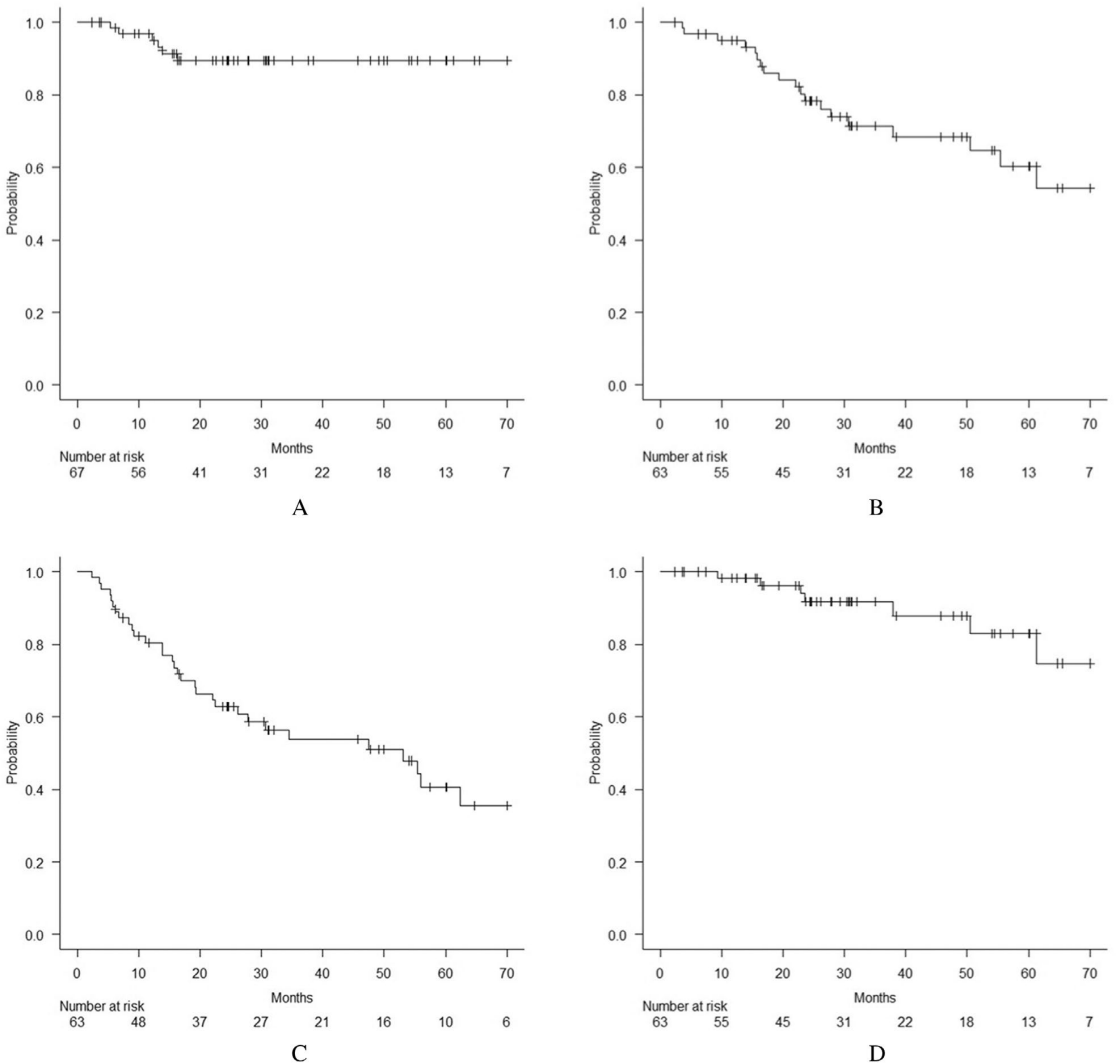
Clinical outcomes after radiotherapy in all eligible patients and tumors. A. Local control (LC) for all eligible tumors (*n* = 67). Three‐ and five‐year LC rates were 89.4% and 89.4%, respectively. B. Overall survival (OS) of all eligible patients (*n* = 63). Median OS was 29.3 months. Three‐ and five‐year OS rates were 71.4% and 60.3%, respectively. C. Progression‐free survival (PFS) of all eligible patients (*n* = 63). Median PFS was 24.6 months. Three‐ and five‐year PFS rates were 53.8% and 40.5%, respectively. D. Disease‐specific survival (DSS) of all eligible patients (*n* = 63). Median DSS was 29.3 months. Three‐ and five‐year DSS rates were 91.8% and 83.1%, respectively.

The median follow‐up was 29.3 months (range: 2.4–120.5), during which we observed six local recurrences; nine ipsilateral pulmonary, five contralateral pulmonary, six lymph node, and six distant metastases; and 20 deaths. LC, OS, PFS, and DSS in all eligible tumors and patients are shown in Figure 1. In terms of adverse events, 10 (15.9%), and four (6.3%) patients experienced pneumonitis grade ≥ 2, and ≥ 3, respectively, including one patient developing grade 4 (1.6%). In addition, two patients developed grade 2 rib fracture 29.9 and 36.6 months after SBRT, respectively. No grade 5 toxicities were observed in this study.

### 3.1 Prognostic Factors After SBRT

The results of univariate analyses that assessed potential prognostic factors for LC, OS, and PFS are listed in Table 2. Using univariate analyses, Sq, D_50_ < 120 Gy_BED10_, D_95_ < 105 Gy_BED10_, and D_max_ < 125 Gy_BED10_ for PTV were associated with worse LC. Regarding the multivariate analyses, Sq was significantly associated with worse LC (Table 3). Patients with Sq, cT1b or c, CTR > 0.25, and PTV ≥ 30 cm^3^ tumors were associated with worse PFS in comparison with those with non‐Sq, ≤ cT1a, CTR ≤ 0.25, PTV < 30 cm^3^ tumors. The PTV ≥ 30 cm^3^ group showed significantly more patients with cT1b–1c tumors and adding CTV margin (*p* = 0.001 and 0.006, respectively) but no significant differences in the incidence of Sq and Dmax_BED10_ ≥ 125 Gy (*p* = 0.060, and 0.188, respectively) in comparison with the PTV < 30 cm^3^ group. In addition, no significant prognostic factors were observed for OS in this study. In addition, Sq was associated with worse LC and PFS using univariate analyses among patients with pathological diagnosis (Supporting Information 2). No significant differences were found in the CTR > 0.25 and ≤ 0.25 groups including patients with pathological diagnosis (*n* = 2 in the ≤ 0.25 group).

**TABLE 2:** Univariate analyses for the potential prognostic factors.

Factors	*N*	(%)	Three‐year rates	*p*‐value
*Local control (n = 67)*				
Age (y)				
< 75	27	40.3%	86.5%	0.622
≥ 75	40	59.7%	91.4%	
Sex				
Female	41	61.2%	94.4%	0.275
Male	26	38.8%	86.4%	
ECOG‐PS				
0, 1	53	79.1%	88.8%	0.751
2−	14	20.9%	91.7%	
Pathological diagnosis				
Sq	12	17.9%	63.6%	0.001
Other	55	82.1%	95.3%	
Clinical T stage				
≤ T1a	16	23.9%	91.7%	0.636
T1b–c	51	76.1%	88.7%	
CTR				
≤ 0.25	5	7.5%	100.0%	0.434
> 0.25	62	92.5%	88.3%	
CTV margin				
Yes	32	47.8%	82.9%	0.097
No	35	52.2%	96.2%	
Volume of PTV				
< 30 cm^3^	35	52.2%	89.6%	0.908
≥ 30 cm^3^	32	47.8%	89.3%	
D_max_ (BED_10_) for PTV				
< 125 Gy	23	34.3%	78.3%	0.017
≥ 125 Gy	44	65.7%	96.8%	
D50 (BED_10_)				
< 120 Gy	37	55.2%	82.2%	0.036
≥ 120 Gy	30	44.8%	100.0%	
D95 (BED_10_)				
< 105 Gy	28	41.8%	80.7%	0.037
≥ 105 Gy	39	58.2%	96.7%	
D98 (BED_10_)				
< 100 Gy	24	35.8%	85.7%	0.445
≥ 100 Gy	43	64.2%	91.6%	
D_min_ (BED_10_)				
< 90 Gy	30	44.8%	90.7%	0.702
≥ 90 Gy	37	55.2%	88.1%	
*Overall survival (n = 63)*				
Age (y)				
< 75	24	38.1%	81.2%	0.594
≥ 75	39	61.9%	66.5%	
Sex				
Female	25	39.7%	70.0%	0.772
Male	38	60.3%	72.1%	
ECOG‐PS				
0, 1	49	77.8%	75.8%	0.136
2−	14	22.2%	46.4%	
Pathological diagnosis				
Sq	12	19.0%	54.9%	0.321
Other	51	81.0%	75.4%	
Clinical T stage				
≤ T1a	13	20.6%	90.0%	0.082
T1b–c	50	79.4%	66.4%	
CTR				
≤ 0.25	5	7.9%	100.0%	0.120
> 0.25	58	92.1%	68.8%	
CTV margin				
Yes	31	49.2%	67.9%	0.746
No	32	50.8%	74.8%	
Volume of PTV				
< 30 cm^3^	32	50.8%	79.3%	0.089
≥ 30 cm^3^	31	49.2%	63.4%	
D_max_ (BED_10_) for PTV				
< 125 Gy	21	33.3%	72.4%	0.439
≥ 125 Gy	42	66.7%	71.0%	
D50 (BED_10_)				
< 120 Gy	35	55.6%	71.9%	0.629
≥ 120 Gy	28	44.4%	70.3%	
D95 (BED_10_)				
< 105 Gy	27	42.9%	64.3%	0.703
≥ 105 Gy	36	57.1%	76.5%	
D98 (BED_10_)				
< 100 Gy	23	36.5%	66.5%	0.777
≥ 100 Gy	40	63.5%	73.6%	
D_min_ (BED_10_)				
< 90 Gy	28	44.4%	74.3%	0.991
≥ 90 Gy	35	55.6%	69.2%	
*Progression‐free survival (n = 63)*				
Age (y)				
< 75	24	38.1%	59.6%	0.620
≥ 75	39	61.9%	52.0%	
Sex				
Female	25	39.7%	57.9%	0.195
Male	38	60.3%	50.3%	
ECOG‐PS				
0, 1	49	77.8%	57.2%	0.464
2−	14	22.2%	37.1%	
Pathological diagnosis				
Sq	12	19.0%	26.7%	0.049
Other	51	81.0%	60.8%	
Clinical T stage				
≤ T1a	13	20.6%	81.5%	0.004
T1b–c	50	79.4%	46.4%	
CTR				
≤ 0.25	5	7.9%	100.0%	0.038
> 0.25	58	92.1%	49.6%	
CTV margin				
Yes	31	49.2%	53.0%	0.777
No	32	50.8%	53.7%	
Volume of PTV				
< 30 cm^3^	32	50.8%	68.7%	0.004
≥ 30 cm^3^	31	49.2%	39.4%	
D_max_ (BED_10_) for PTV				
< 125 Gy	21	33.3%	51.4%	0.874
≥ 125 Gy	42	66.7%	55.2%	
D50 (BED_10_)				
< 120 Gy	35	55.6%	52.0%	0.949
≥ 120 Gy	28	44.4%	57.0%	
D95 (BED_10_)				
< 105 Gy	27	42.9%	47.7%	0.418
≥ 105 Gy	36	57.1%	58.3%	
D98 (BED_10_)				
< 100 Gy	23	36.5%	48.7%	0.883
≥ 100 Gy	40	63.5%	56.3%	
D_min_ (BED_10_)				
< 90 Gy	28	44.4%	51.5%	0.614
≥ 90 Gy	35	55.6%	54.8%	

Abbreviations: BED, biologically effective dose; CTR, Consolidation tumor ratio; CTV, clinical target volume; D_max_, the maximum dose; D_min_, the minimum dose; Dxx, the minimum dose received by XX% of the total volume of PTV; ECOG‐PS, Eastern Cooperative Oncology Group performance status; PTV, planning target volume; Sq, Squamous cell carcinoma.

**TABLE 3:** Multivariate analyses for the potential prognostic factors.

Factors	*n*	(%)	Hazard ratio (95% CI)	*p*‐value
*Local control (n = 67)*				
Pathological diagnosis				
Sq	12	17.9%	1	0.012
Other	55	82.1%	0.112 (0.020–0.617)	
D_max_ (BED_10_) for PTV				
< 125 Gy	23	34.3%	1	0.066
≥ 125 Gy	44	65.7%	0.133 (0.015–1.147)	
*Progression‐free survival (n = 63)*				
Pathological diagnosis				
Sq	12	19.0%	1	0.619
Other	51	81.0%	0.810 (0.353–1.858)	
Clinical T stage				
≤ T1a	13	20.6%	1	0.165
T1b–c	50	79.4%	2.882 (0.648–12.830)	
CTR				
≤ 0.25	5	7.9%	1	0.997
> 0.25	58	92.1%	Not applicable	
Volume of PTV				
< 30 cm^3^	32	50.8%	1	0.129
≥ 30 cm^3^	31	49.2%	1.870 (0.833–4.199)	

Abbreviations: BED, biologically effective dose; CI, confidence interval; CTR, Consolidation tumor ratio; D_max_, the maximum dose; PTV, planning target volume; Sq, Squamous cell carcinoma.

### 3.2 Impact of GGO Predominance in Prognosis After SBRT

**FIGURE 2:**
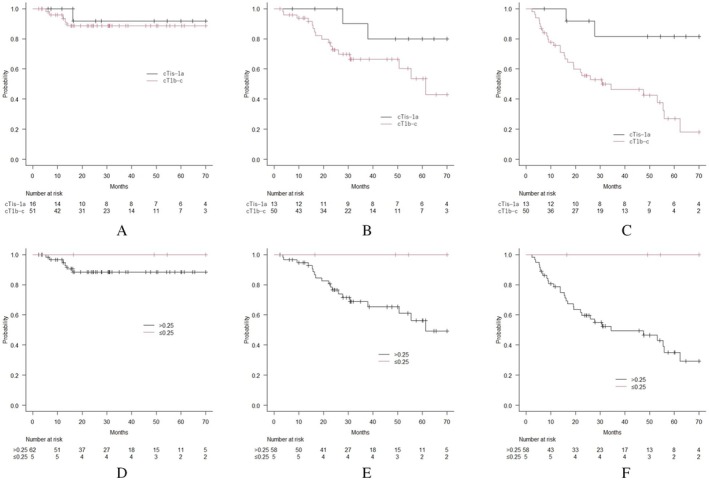
Clinical outcomes per T stage and consolidation tumor ratio in all eligible patients and tumors. A. Local control (LC) for tumors per T stage. No significant differences were observed between the two groups (*n* = 16 and 51 in cT1a or less and cT1b and c groups, respectively; *p* = 0.636). B. Overall survival (OS) of patients per T stage. No significant differences were observed between the two groups (*n* = 13 and 50 in cT1a or less and cT1b and c groups, respectively; *p* = 0.082). C. Progression‐free survival (PFS) of patients per T stage. Patients with tumors of cT1a or less showed significantly better PFS than those with cT1b or c (*n* = 13 and 50 in cT1a or less and cT1b and c groups, respectively; *p* = 0.004). D. LC for tumors per consolidation tumor ratio (CTR). No significant differences were observed between the two groups (*n* = 5 and 62 in CTR ≤ 0.25 and > 0.25 groups, respectively; *p* = 0.434). E. OS for patients per CTR. No significant differences were observed between the two groups (*n* = 5 and 58 in CTR ≤ 0.25 and > 0.25 groups, respectively; *p* = 0.120). F. PFS for patients per CTR. Patients with tumors of CTR ≤ 0.25 showed significantly better PFS than those with > 0.25 (*n* = 5 and 58 in CTR ≤ 0.25 and > 0.25 groups; *p* = 0.038).

LC, OS, and PFS between the T1a or less and T1b or c groups, and also between CTR ≤ 0.25 and > 0.25 groups are shown in Figure 2. There were no recurrences, metastases, or deaths in patients with tumors of CTR ≤ 0.25 (*n* = 5). Sixteen patients in the T1a or less group included five patients with CTR ≤ 0.25. Patients with T1a or less, and with CTR ≤ 0.25 had significantly better PFS than did those with T1b–c or with CTR > 0.25 (Figure 2C,F). In addition, no significant differences were observed in LC and OS between the T1a or less and T1b–c group (Figure 2A,B) and also the CTR ≤ 0.25 and > 0.25 groups (Figure 2D,E).

**FIGURE 3:**
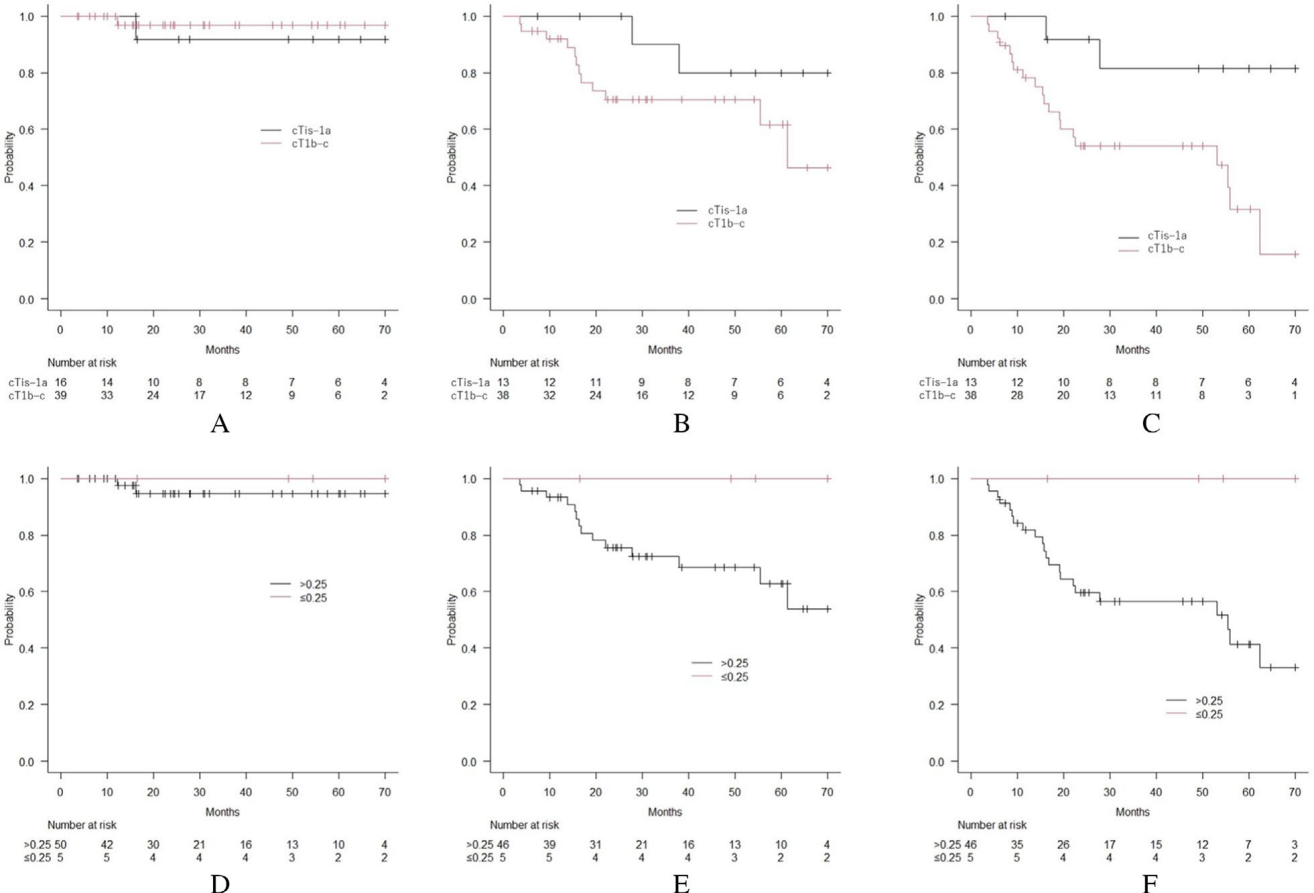
Clinical outcomes a per T stage and consolidation tumor ratio in patients and tumors excluding squamous cell carcinoma. A. Local control (LC) for tumors per T stage. No significant differences were observed between the two groups (*n* = 16 and 39 in cT1a or less and cT1b and c groups, respectively; *p* = 0.533). B. Overall survival (OS) of patients per T stage. No significant differences were observed between the two groups (*n* = 13 and 38 in cT1a or less and cT1b and c groups, respectively; *p* = 0.130). C. Progression‐free survival (PFS) of patients per T stage. Patients with tumors of cT1a or less showed significantly better PFS than those with cT1b or c (*n* = 13 and 38 in cT1a or less and cT1b and c groups, respectively; *p* = 0.008). D. LC for tumors per consolidation tumor ratio (CTR). No significant differences were observed between the two groups (*n* = 5 and 50 in CTR ≤ 0.25 and > 0.25 groups, respectively; *p* = 0.602). E. OS for patients per CTR. No significant differences were observed between the two groups (*n* = 5 and 46 in CTR ≤ 0.25 and > 0.25 groups, respectively; *p* = 0.146). F. PFS for patients per CTR. Patients with tumors of CTR ≤ 0.25 trended to have better PFS than those with > 0.25 (*n* = 5 and 46 in CTR ≤ 0.25 and > 0.25 groups; *p* = 0.057).

In 51 patients with 55 tumors and excluding patients with Sq tumors, a significant difference was found in PFS between the T1a or less and T1b–c groups; the CTR ≤ 0.25 group tended to show better PFS than did the > 0.25 group (Figure 3C). In addition, no significant differences were observed in LC and OS between the T1a or less and T1b–c groups (Figure 3A,B) and also the CTR ≤ 0.25 and > 0.25 groups (Figure 3D,E).

Among the 11 patients with 11 cT1a tumors, no significant differences were found in LC, OS, and PFS between patients with pure solid tumors (CTR = 1) and those with GGO component (CTR < 1) (*p* = 0.527, 0.897, and 0.941, respectively).

## 4 Discussion

This study sought to assess the potential prognostic factors and investigate the prognostic impact of the CTR and tumor size on treatment outcomes in patients with stage 0–IA NSCLC treated with SBRT. Our findings showed that SBRT could be a safe and effective treatment of medically inoperable patients with GGO‐predominant tumors. Importantly, patients with tumors with CTR ≤ 0.25, corresponding to Tis and T1mi according to the UICC 8th edition, developed no failures after SBRT and showed significantly better PFS than did those with CTR > 0.25. These findings suggested a correlation between low CTR and favorable prognosis corresponding with those undergoing surgical resection [5].

In the present study, elevated dose, D_50_, D_95_, and D_max_ for PTV of < 120 Gy_BED10_, < 105 Gy_BED10_, and < 125 Gy_BED10_ and the Sq tumor demonstrated a trend toward higher rates of local recurrence in patients with cTis‐1 tumors. In addition, the Sq group tended to show worse PFS, which might be due to poorer LC. There are only a few reports documenting the benefit of elevated doses for PTV in cTis‐1 tumor cases, which have a potentially better LC than do clinical T2 tumors [20]. This study showed that the elevated D_50_, D_95_, and D_max_ groups tended to have a better LC than did the lower doses group in patients with Tis‐1 tumors; however, no significant differences were observed using the multivariate analysis due to the limited sample size. In a recent phase 3 clinical trial, the JCOG 1408 study, a dose of 42 Gy was applied in four fractions prescribed to the D_95%_ of the PTV at 80% isodose lines meaning D_2%_ ≥ 121 Gy_BED10_, in the standard dose group [27]. D_max_ ≥ 125Gy seemed to correspond to D_2%_ ≥ 121 Gy_BED10_. In addition, elevated PTV maximum dose has been recommended in terms of SBRT in the modern era [31, 32]. Therefore, elevated doses such as D_max_ ≥ 125 Gy_BED10_ for PTV seem reasonable in the modern SBRT strategy for cTis–1 NSCLC.

We have previously reported that Sq was associated with poorer LC in patients with cT1‐2 tumors treated with SBRT [20], which is consistent with the findings in this study. Sq has been shown to be associated with a higher risk of recurrence following SBRT, potentially due to differences in radiosensitivity compared with adenocarcinomas [33]. In addition, we previously demonstrated that delivering elevated central doses could overcome the inferior LC in Sq tumors [20]. Sub‐analysis assessing the elevated doses for PTV among the Sq tumors was not performed in this study because all Sq tumors received ≤ 125 Gy_BED10_ for PTV. However, the Sq tumor was an independent predictive factor for worse LC in the multivariate analysis and showed a tendency of worse PFS in both the present study and that previous study [20].

In this study, cTis‐1a, CTR ≤ 0.25, and the volume of PTV < 30 cm^3^ were associated with better PFS; however, there were no significant differences between the two groups using multivariate analysis. Prognostic factors for PFS after SBRT in patients with NSCLC have been poorly reported in previous studies [21, 34]. The PTV ≥ 30 cm^3^ group included more patients with cT1b–1c tumors and additional CTV margin. Thus, more advanced tumor stage and the limited clinical benefit of CTV margin in SBRT might lead to inferior PFS in the PTV ≥ 30 cm^3^ compared with the < 30 cm^3^ group. We have previously reported that clinical T stage was associated with PFS among patients with cT1 to 3 lung cancer [21]. Duijm et al. have reported that PTV volume and clinical stage (IA‐IIA vs. IIB‐IIIA) were associated with disease progression [34]. The findings in this study are consistent with those in previous reports [21, 34]. However, to the best of our knowledge, there are no reports focusing on prognostic factors for PFS in Tis‐1 tumors. Adjuvant immune checkpoint inhibitor (ICI) following SBRT for cN0 early‐stage NSCLC has been recently studied in clinical trials [35, 36]. However, most eligible patients who could be enrolled in clinical trials assessing ICI plus SBRT for cN0 NSCLC have a more advanced stage than this study, i.e., cT1‐3 [35]. In light of the present data, tumors of cT1b or greater and relatively large tumors might benefit from consolidation treatment with an adjuvant strategy following SBRT for early‐stage NSCLC.

Patients with GGO‐predominant tumors, CTR ≤ 0.25, developed no failures after SBRT and showed a significantly better PFS than did those with CTR > 0.25. In addition, the CTR ≤ 0.25 group showed a tendency of better PFS, except in those with Sq tumors. The integration of CTR into the TNM staging system, as introduced in the 8th edition of the UICC classification, represents an advancement in predicting the biological behavior of lung cancers [4]. Tumors with low CTR are often predominantly composed of GGO and typically associated with less aggressive histological subtypes such as adenocarcinoma in situ or minimally invasive adenocarcinoma, which generally have a more indolent clinical course [5]. Suzuki et al. have reported favorable outcomes, five‐year relapse‐free survival of 99.7% in patients with early‐stage NSCLC with wedge resection along with 5.4% of grade ≥ 3 toxicities [5]. Our study emphasizes that in the CTR ≤ 0.25 cohort, which aligns with the Tis/T1mi stage in the UICC 8th edition, there were no observed failures, further reinforcing the role of CTR as a predictor of favorable outcomes in patients with early‐stage NSCLC. These results are consistent with those in prior studies that have demonstrated the efficacy of SBRT in patients with GGO‐predominant tumors, with high rates of LC and minimal toxicity [22, 24]. Previous surgical series have established that wedge resection of tumors with CTR < 0.25 and segmentectomy of tumors with CTR < 0.5 were associated with high survival rates and low recurrence [5, 6]. Those results seem to indicate less invasive strategies that can be optimal for radical treatment of early‐stage NSCLC. In terms of adverse events, the incidence of grade ≥ 2 and ≥ 3 radiation pneumonitis were 15.9% and 6.3%, respectively. The incidence of symptomatic and severe toxicity seems compatible with minimally invasive surgical and SBRT series [5, 6, 9]. In addition, SBRT does not require anesthesia, post‐procedural complications are rare, and less likely to lead to infection or bleeding in comparison with surgery. Therefore, we suggest that the role of SBRT for GGO‐predominant NSCLC should be explored further. However, the role of histological subtype, dose escalation, and patient‐related factors must be considered when determining the optimal treatment strategy. Future studies comparing SBRT with surgical resection in this subgroup will offer valuable insights into the optimal management approach in patients with low CTR tumors.

Naturally, our study has several limitations, including the retrospective, single‐center design with a relatively small sample size and short follow‐up term, which might limit the generalizability of our findings. SBRT is well established in medically inoperable patients such as older adults, those with a high prevalence of comorbidities, and those in poor general condition. Nonetheless, in this study, all eligible tumors were assessed in the same manner by HRCT. In addition, favorable PFS was validated in the cTis‐1a and CTR ≤ 0.25 groups except for patients with Sq tumors; however, no significant differences were found between the CTR ≤ 0.25 and > 0.25 groups, perhaps due to the limited sample size, particularly the CTR ≤ 0.25 group, which included five patients. The optimal cut‐off point for CTR in terms of SBRT for early‐stage NSCLC is unclear. However, the cut‐off point of 0.25 was previously confirmed to be a predictor for identifying patients with early‐stage NSCLC undergoing surgery in a prospective study and has been applied in a prospective clinical trial [5, 29]. Therefore, we considered that CTR of 0.25 was a reasonable cut‐off point to assess early‐stage NSCLC. Conversely, patients with tumors with a GGO component tend to have better survival in an analysis of patients with cT1a tumors in a surgical series [37]. The optimal cut‐off point for CTR should be further assessed in future studies including a large number of patients undergoing SBRT. The cohort predominantly included older patients, many of whom had underlying health conditions that might have negatively influenced their survival. A three‐year DSS of 91.8% in patients with a median age of 77 years is consistent with previous surgical and SBRT series [9, 38]. Furthermore, this study included 52.2% of tumors undergoing SBRT without pathological diagnosis. Previous studies showed the percentages of non‐pathological confirmation cases ranging between 29% and 65% [39]. The proportion of cases without biopsy seems to be correspondent with the previous studies. The impact of misclassification bias on outcomes is poorly understood. The high rate of absence of pathological diagnosis might be associated with an inferior survival outcome in this study [40]. However, Fan et al. reported that no significant differences were observed between the clinical and pathological diagnosis cohorts in terms of the long‐term outcome after SBRT in a retrospective study [41]. Thus, further studies are needed to assess patient selection for SBRT for small lung tumors without pathological confirmation, as this might lead to overtreatment. In addition, the median follow‐up period of 29.3 months seems sufficient to assess LC and survival outcomes, with consideration of the median age; however, the follow‐up period might not fully capture long‐term survival outcomes [9]. Larger, multi‐center longitudinal studies are needed to further validate the role of CTR in predicting treatment outcomes and assess the potential for SBRT as an alternative to surgery in select patients with early‐stage, GGO‐predominant lung cancers.

In conclusion, patients with early‐stage NSCLC with cTis‐1a could archive excellent oncological outcomes after SBRT. In addition, NSCLC with a low CTR of ≤ 0.25 seemed to have a low risk of recurrence after SBRT. The findings in this study support the use of SBRT for GGO‐predominant early‐stage NSCLC and raise the prospect of comparing SBRT to surgical resection in future studies. Further investigation through prospective, multi‐center trials with larger patient cohorts is needed to assess the potential for SBRT to be an alternative to surgery for selected patients with early‐stage, GGO‐predominant NSCLC.

## Author Contributions

Conceptualization, H.D.; Methodology, J.F. and H.D.; Software, J.F.; Validation, J.F., H.D., and Y.M.; Formal Analysis, J.F.; Investigation, J.F., H.D., A.K., T.I., S.T.I., N.I.H., T.U., M.I., K.N., H.H., K.I., and Y.M.; Resources, J.F. and H.D.; Data Curation, J.F., A.K., T.I., and M.I.; Writing – Original Draft Preparation, J.F. and H.D.; Writing – Review and Editing, H.D., A.K., and Y.M.; Visualization, J.F.; Supervision, K.I. and Y.M.; Project Administration, J.F. and H.D.; Funding Acquisition, H.D. All the authors read and approved the final manuscript.

## Ethics Statement

This study was conducted according to the guidelines of the Declaration of Helsinki. This study was approved by our institutional review board with approval number R06‐173.

Informed Consent Statement: Written informed consent for radiotherapy was obtained from all individual participants prior to radiotherapy. Informed consent for this study was obtained in the form of opt‐out.

## Consent

The requirement for informed consent was waived for this retrospective study.

## Conflicts of Interest

H.D. received speaker fees from AstraZeneca, Eisai, Boston Scientific Japan, Varian Medical Systems, and Accuray Japan K.K. Y.M. received speaker fees from AstraZeneca and Varian Medical Systems and has been a member of the guideline committee of the Japan Lung Cancer Society. All other authors declare no conflicts of interest.

## Supporting information


**Data S1.** Supporting Information.

**Data S2.** Supporting Information.

## Data Availability

The data are available from the corresponding authors upon reasonable request.
