# Supplementary material for: Prognostic Impact of Tumor Solid Components in Stereotactic Body Radiotherapy for Clinical Stage Tis–1N0M0 Lung Cancer
Source: Thorac Cancer. 2025 Jun 16;16(11):e70110. doi: 10.1111/1759-7714.70110 (PMC12168223; doi:10.1111/1759-7714.70110)
Supplement: Supplementary file 1 — Data S1. Supporting Information. [file TCA-16-e70110-s001.pdf]

Supplementary data 1. Flowchart outlining the selection of patients.

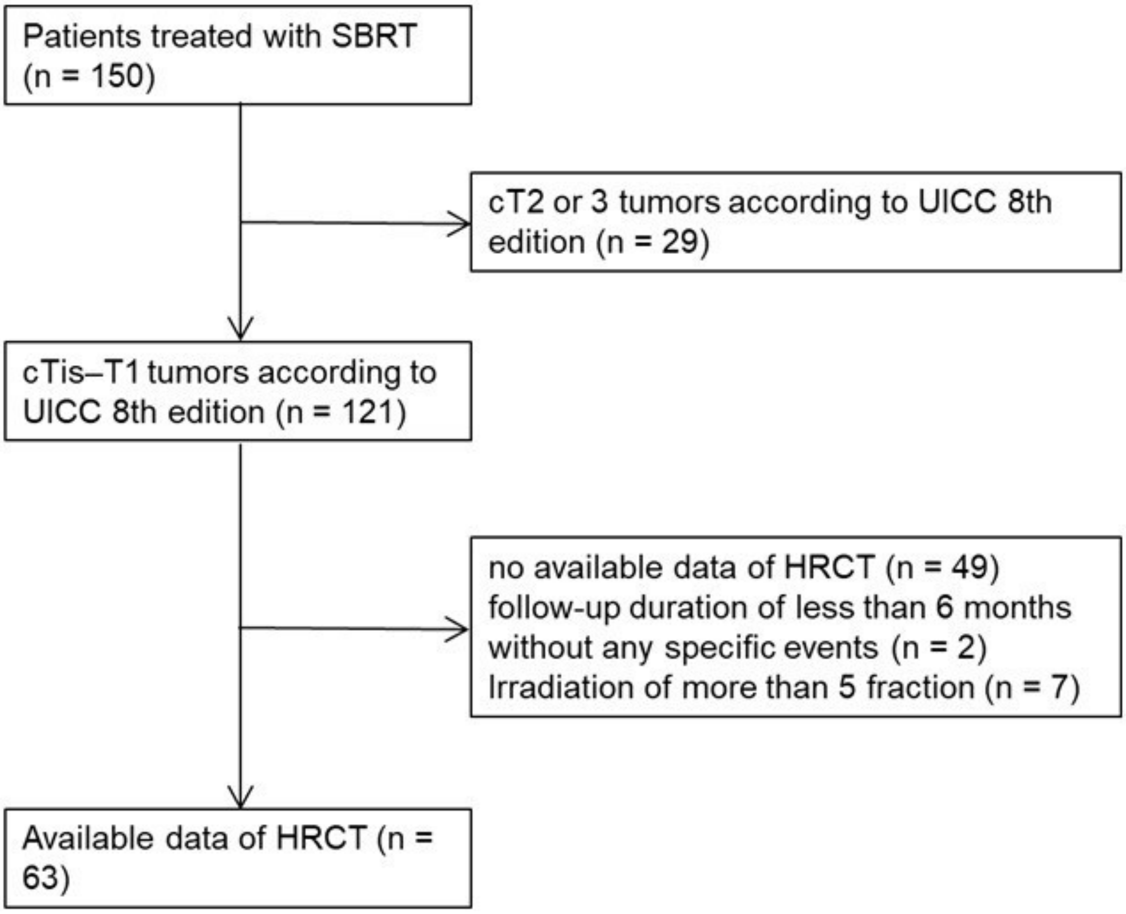

A total of **63 consecutive patients with 67 lesions of clinical stage 0–IA lung cancer** which was diagnosed according to UICC 8th edition using HRCT were retrospectively analyzed in this study.

Abbreviations: SBRT, stereotactic body radiotherapy; UICC, Union for International Cancer Control; HRCT, high-resolution computed tomography.
