# Supplementary material for: Prognostic Impact of Tumor Solid Components in Stereotactic Body Radiotherapy for Clinical Stage Tis–1N0M0 Lung Cancer
Source: Thorac Cancer. 2025 Jun 16;16(11):e70110. doi: 10.1111/1759-7714.70110 (PMC12168223; doi:10.1111/1759-7714.70110)
Supplement: Supplementary file 2 — Data S2. Supporting Information. [file TCA-16-e70110-s002.pdf]

Supplementary data 2. Univariate analyses for the potential prognostic factors among patients with pathological diagnosis.

Factors	n	(%)	Three year rates	P-value
**Local control (n = 32)**				
Pathological diagnosis				
Sq	12	37.5%	63.6%	0.031
Other	20	62.5%	94.4%	
CTR				
≤ 0.25	2	6.3%	100.0%	0.529
> 0.25	30	93.8%	81.7%	
**Overall survival (n = 32)**				
Pathological diagnosis				
Sq	12	37.5%	54.9%	0.103
Other	20	62.5%	84.4%	
CTR				
≤ 0.25	2	6.3%	100.0%	0.328
> 0.25	30	93.8%	71.8%	
**Progression-free survival (n = 32)**				
Pathological diagnosis				
Sq	12	37.5%	26.7%	0.042
Other	20	62.5%	68.8%	
CTR				
≤ 0.25	2	6.3%	100.0%	0.137
> 0.25	30	93.8%	49.5%	

Abbreviations: Sq, Squamous cell carcinoma; CTR, Consolidation tumor ratio.
